# The role of Tg kinetics in predicting 2-[^18^F]-FDG PET/CT results and overall survival in patients affected by differentiated thyroid carcinoma with detectable Tg and negative 131I-scan

**DOI:** 10.1007/s12020-021-02755-5

**Published:** 2021-05-20

**Authors:** Domenico Albano, Mark Tulchinsky, Francesco Dondi, Angelica Mazzoletti, Francesco Bertagna, Raffaele Giubbini

**Affiliations:** 1 Nuclear Medicine, University of Brescia and Spedali Civili Brescia, Brescia, Italy; 2 Section of Nuclear Medicine, Department of Radiology, Milton S. Hershey Medical Center, Penn State Health, Hershey, PA USA

**Keywords:** Thyroglobulin doubling time, Thyroglobulin velocity, Thyroglobulin kinetics, Differentiated thyroid carcinoma, 2-[^18^F]-FDG PET/CT

## Abstract

**Purpose:**

The aim of this study was to assess the potential role of thyroglobulin (Tg) kinetics in predicting 2-[^18^F]-FDG-PET/CT results and overall survival (OS) in patients affected by differentiated thyroid carcinoma (DTC) and suspected recurrence.

**Methods:**

On hundred and thirty-nine patients were retrospectively included. All patients underwent 2-[^18^F]-FDG-PET/CT due to detectable Tg levels and negative [^131^I] whole-body scan. The last two consecutive serum Tg measurements before PET/CT were used for Tg-doubling time (TgDT) and Tg-velocity (Tg-vel) calculation. Receiver operating characteristic (ROC) curves were used to determine the optimal cutoff points for Tg, TgDT and Tg-vel for predicting PET/CT results.

**Results:**

One hundred and fifteen (83%) patients had positive PET/CT for DTC recurrence, while the remaining 24 (17%) negative. Stimulated Tg before PET and Tg-vel were significantly higher in patients with a positive PET/CT scan than negative scan (average Tg 190 vs 14 ng/mL, *p* = 0.006; average Tg-vel 4.2 vs 1.7 ng/mL/y, *p* < 0.001). Instead, TgDT was significantly shorter in positive scan (average TgDT 1.4 vs 4.4 years, *p* < 0.001). ROC curve analysis revealed the best Tg, TgDT and Tg-vel cutoff of 18 ng/mL,1.36 years and 1.95 ng/mL/y. In patients with Tg<18 ng/mL, the PET/CT detection rate was significantly lower in patients with low Tg-vel (*p* = 0.018) and with long TgDT (*p* = 0.001). ATA class risk, PET/CT results and Tg before PET were confirmed to be independent prognostic variables for OS.

**Conclusions:**

Tg kinetics may help to predict 2-[^18^F]-FDG-PET/CT results in DTC patients with negative [^131^I]WBS and detectable Tg, especially in case of low-moderate Tg.

## Introduction

Differentiated thyroid cancer (DTC) is the most diffuse endocrine cancer and it is usually considered a cancer with favorable management and a long-term survival [1], except of the cases with distant metastases (especially bone) and iodine refractory disease [2].

Excluding low-risk disease, the baseline treatment of DTC consists of thyroidectomy followed by postoperative risk-adapted sodium iodide ([^131^I]) therapy if indicated [1, 3]. This approach leads to an excellent response in the majority [1, 4], often even 6–12 months after primary therapy [5]. However, a long-term active follow-up is mandatory because DTC is a dynamic disease and the possibility to have persistent disease or relapse is not uncommon, especially in intermediate and high risk patients [6].

In this clinical scenario, a serum marker useful to check the DTC disease status and its evolution is fundamental. Serum thyroglobulin (Tg) is a sensitive tumor marker applied in the detection of residual disease and/or surveillance for recurrent DTC [7]. Neck ultrasound and diagnostic [^131^I] whole-body scan ([^131^I]WBS) are the most accurate tools to localize and stage/restage DTC [1], but they have some limitations. For example, Tg is interfered by the presence of detectable Tg-antibodies that reduces its sensitivity, while [^131^I]WBS is able to detect only iodine-avid disease.

The 2-deoxy-2-[^18^F]fluoro-D-glucose positron emission tomography/computed tomography (2-[^18^F]-FDG PET/CT) is a non-invasive tool able to identify non-iodine avid disease, particularly in patients with detectable or increasing Tg levels who had a negative [^131^I]WBS and it is useful to define the prognosis and the presence of RAI-refractory disease [8–10].

There is a wide range of proposed Tg thresholds to achieve optimal 2-[^18^F]-FDG PET/CT accuracy in patients affected by DTC [8, 11–13]. This varied interval is probably due to the intrinsic limitations of Tg as “one-shot” absolute value: such as that serum Tg levels are for definition dependent on circulating thyrotropin (TSH) concentrations, the different stimulated and unstimulated threshold proposed in literature and the absence of the evaluation of Tg trend in time.

Few studies suggested a prognostic role of post-ablation Tg-doubling time (TgDT) in predicting survival outcome [14–20], while only two papers demonstrated [21, 22] that TgDT was an independent factor to predict 2-[^18^F]-FDG PET/CT results.

Instead, considering Tg-velocity (Tg-vel), only one paper is available [23].

The aim of this study was to assess the potential usefulness of Tg kinetics (Tg, TgDT, Tg-vel) in predicting 2-[^18^F]-FDG PET/CT results and to compare their relative efficacy for restaging DTC patients with detectable Tg and a negative [^131^I]WBS.

## Materials and methods

### Patients selection

We retrospectively screened 2500 patients who underwent [^131^I] therapy for DTC after total or nearly total thyroidectomy from December 2006 to December 2020 using our institutional Radiology Information System. They were admitted to our Nuclear Medicine Department for the ablation of thyroid remnant and/or therapy according to EANM (European Association of Nuclear Medicine) guidelines [24]. The administered activity of ^131^I first ablation treatment was established according to the risk class based on the TNM staging of the American Joint Committee on Cancer/International Union against Cancer currently in use and the status of the disease. Inclusion criteria were (1) the presence of a 2-[^18^F]-FDG PET/CT scan, (2) negative near [^131^I]WBS, (3) two consecutive Tg measurements under the thyroid hormone replacement therapy to calculate Tg kinetics before 2-[^18^F]-FDG PET/C; (4) no interfering antiTg antibodies.

### 2-[^18^F]-FDG PET/CT imaging and interpretation

2-[^18^F]-FDG PET/CT scans were performed following the EANM guidelines [25]. All patients underwent 2-[^18^F]-FDG PET/CT after at least 6 h of fasting and with blood glucose level lower than 150 mg/dL. An activity of 3.5–4.5 MBq/Kg of radiotracer was administered intravenously and examinations were acquired about 60 ± 10 min after injection from the skull base to the mid-thigh on a Discovery 690 or DST PET/CT tomograph (General Electric Company—GER—Milwaukee, USA) with standard parameters (CT: 80 mA, 120 kV without contrast; 2.5–3.5 min per bed-PET-step of 15 cm); the reconstructions were performed in a 128 × 128 or 256 × 256 matrix and 60 cm field of view.

2-[^18^F]-FDG PET/CT were performed during hospitalization for the radiometabolic therapy with 131I to study the FDG-avidity of DTC localizations and compared these findings with the previous or concomitant [131I]WBS.

The PET/CT images were reviewed by two expert nuclear medicine physicians (DA, FB) and every focal tracer uptake different from physiological background and with activity higher than the surrounding tissue was considered as indicative of disease. PET/CT results was compared with a combination of clinical and/or imaging follow-up for at least 6 months taken as reference standard, including cytological reports, histopathologic reports and further imaging studies such as ultrasound, CT with or without contrast, magnetic resonance imaging and/or subsequent PET/CT. In addition, the rising Tg trend over 6 months was accepted as a biochemical indication of disease. Due to the fact that histopathological confirmation of all lesions detected by PET/CT was not ethically and clinically feasible in every patient, the histological confirmation was available in 53 patients where it was warranted.

The mean follow-up time was 44 months (range 2–173 months).

### Thyroglobulin evaluation

Tg was measured using an immunoradiometric assay (DYNOtest^®^ Tg-plus; BRAHMS Diagnostica, Hennigsdorf, Germany) according to the manufacturer’s instructions. The presence of autoantibodies against Tg was evaluated by a specific radioimmunoassay (DYNOtest^®^ anti-Tg_n_; BRAHMS Diagnostica, Hennigsdorf, Germany).

For our analysis we evaluated the last stimulated Tg prior to 2-[^18^F]-FDG PET/CT (within two months), the Tg-DT and the Tg-velocity. Stimulated Tg before PET/CT was measured after levothyroxine withdrawal (for 40 days, replaced by triiodothyronine in the first 20 days) in 126 patients, and after recombinant human thyrotropin (rhTSH) stimulation in the remaining 13 cases. rhTSH (Genzyme Corporation) was administered intramuscularly with a dose of 0.9 mg on 2 consecutive days during treatment with levothyroxine, and radioiodine was administered the day after the second injection and PET/CT scans performed immediately before radioiodine therapy.

The TgDT was calculated in years on the basis of the last two consecutive Tg levels under TSH suppression therapy obtained prior to the PET/CT scan. This computation was done following procedure by Kuma Hospital (https://www.kuma-h.or.jp/english/about/doubling-time-progression-calculator/). Tg-vel was derived on the basis of the same two consecutive Tg levels under TSH suppression used for Tg-DT and calculated by [(Tg2–Tg1)/(time elapsed in years)] and expressed in ng/mL/y.

### Statistical analysis

Statistical analyses were performed out using MedCalc Software version 18.1 (Belgium). In the descriptive analysis, the categorical variables were represented as simple and relative frequencies, while the numeric variables as mean, median and range values. The detection rate of 2-[^18^F]-FDG PET/CT was evaluated as positive or negative depending on PET/CT findings.

The Tg kinetics measurements (Tg, Tg-vel, and TgDT) were assessed as having normal distribution by the Shapiro–Wilk test. The Mann–Whitney *U* test was used to compare the distribution of variance in different groups. Differences between categorical values were assessed using the two-tailed chi-squared test (*χ*^2^). A *p* value < 0.05 was considered to indicate statistical significance.

For the entire population, receiver operating characteristic (ROC) curve analysis was used and the Youden index was identified to derive the optimal cutoff point of Tg, Tg-vel, and TgDT in the light of which interpret the results of 2-[^18^F]-FDG PET/CT. Subsequently, ROC curves were compared using a nonparametric method described by DeLong.

Overall survival (OS) was calculated from the date of baseline 2-[^18^F]-FDG PET/CT to the date of death from any cause or to the date of last follow-up. Survival curves were plotted according to the Kaplan–Meier method and differences between groups were analyzed by using a two-tailed log rank test. Cox regression was used to estimate the hazard ratio and its confidence interval (CI).

## Results

### Patient features

Finally we recruited 139 DTC patients [*F* = 64, *M* = 75, median age 56 years, range: 20–82 years; male to female ratio = 1.2:1] who underwent 2-[^18^F]-FDG PET/CT with last post therapeutic [^131^I]WBS negative and detectable Tg (≥1 ng/mL) without detectable Tg-antibodies and Tg in increasing. There was a partial overlap (n 80) of patients included with a previous paper of the same center [22].

All patients were primarily treated with total thyroidectomy plus ^131^I with a mean activity of 3.3 GBq (range 1–5.5 GBq). Classic variant of papillary DTC was the most frequent histotype with 50 cases, followed by follicular DTC with 32 cases and aggressive papillary variants in 22 (ten tall cells variant of papillary carcinoma, six sclerosing diffuse variant of papillary carcinoma, five columnar variant of papillary carcinoma, one hobnail variant).

The main clinicopathologic features are resumed in Table 1.

**Table 1:** Baseline features of our population

	Average (range)	Patients *n* (%)
Age years	55.5 (20–82)	
Gender		
Male		75 (54%)
Female		64 (46%)
Histotype		
Papillary		50 (36%)
Follicular variant of papillary		21 (15%)
Follicular		32 (23%)
Aggressive variant		22 (16%)
Hurtle cell		13 (9%)
Unknown (Tx)		1 (1%)
Tumor size (mm)	33 (6–90)	
Multicentricity		33 (24%)
Thyroiditis		10 (7%)
T-stage		
sTx		1 (1%)
sT1		26 (19%)
sT2		31 (22%)
sT3		65 (47%)
sT4		16 (11%)
N-stage		
sN0		84 (60%)
sN1a		29 (21%)
sN1b		26 (19%)
M-stage		
sM1		13 (9%)
ATA class risk		
Low		29 (21%)
Intermediate		81 (58%)
High		29 (21%)
Tg at the time of ablation (ng/mL)	165 (0.1–600)	
TgAb positive at ablation		21 (15%)
First RAI activities administrated (GBq)	3.3 (1–5.5)	
Cumulative RAI activities administrated (GBq)	31.9 (3.7–96.6)	
N° therapies	4.6 (1–10)	

*n* number, *GBq* Gigabequerel, *RAI* radioiodine, *var* variant, *Tg* thyroglobulin, *Ab* antibodies

### Relationship between the detection rate of PET and Tg kinetics in whole population

Among 139 2-[^18^F]-FDG PET/CT, 115 (83%) were interpreted as positive for disease based on showing the presence of increased FDG uptake sites, while the remaining 24 (17%) were negative showing no hypermetabolic lesions in the whole body. The overall [^18^F]FDG PET/CT sensitivity, specificity, PPV, NPV, accuracy, positive and negative likelihood ratios were 97% (95%, CI 92–99%), 82% (95%, CI 66–92%), 94% (95%, CI 89–97%), 91% (95%, CI 77–97%), 93% (95%, CI 88–97%), 5.28 and 0.03, respectively.

The mean stimulated Tg levels at the time of 2-[^18^F]-FDG PET/CT was 159 ng/mL (range 1.1–1600) and was significantly higher in patients with a positive PET/CT study than in patients with a negative scan (mean Tg 190 vs 14 ng/mL, *p* = 0.006).

The mean TgDT and Tg-vel were 1.9 years (range 0.1–29) and 2.1 ng/mL/year (range 0.01–16), respectively. TgDT was significantly shorter in patients with positive PET/CT scan than negative (average TgDT 1.4 vs 4.4 years, *p* < 0.001), and Tg-vel was significantly higher in patients with positive PET/CT scan than negative (average Tg velocity 4.2 vs 1.7 ng/mL/y, *p* < 0.001). The average value of the last two Tg under TSH suppression were 8.7 ng/mL (0.2–289) and 11.6 ng/mL (0.7–1500) with a mean interval of 9.1 months (2–16 months).

**Fig. 1:**
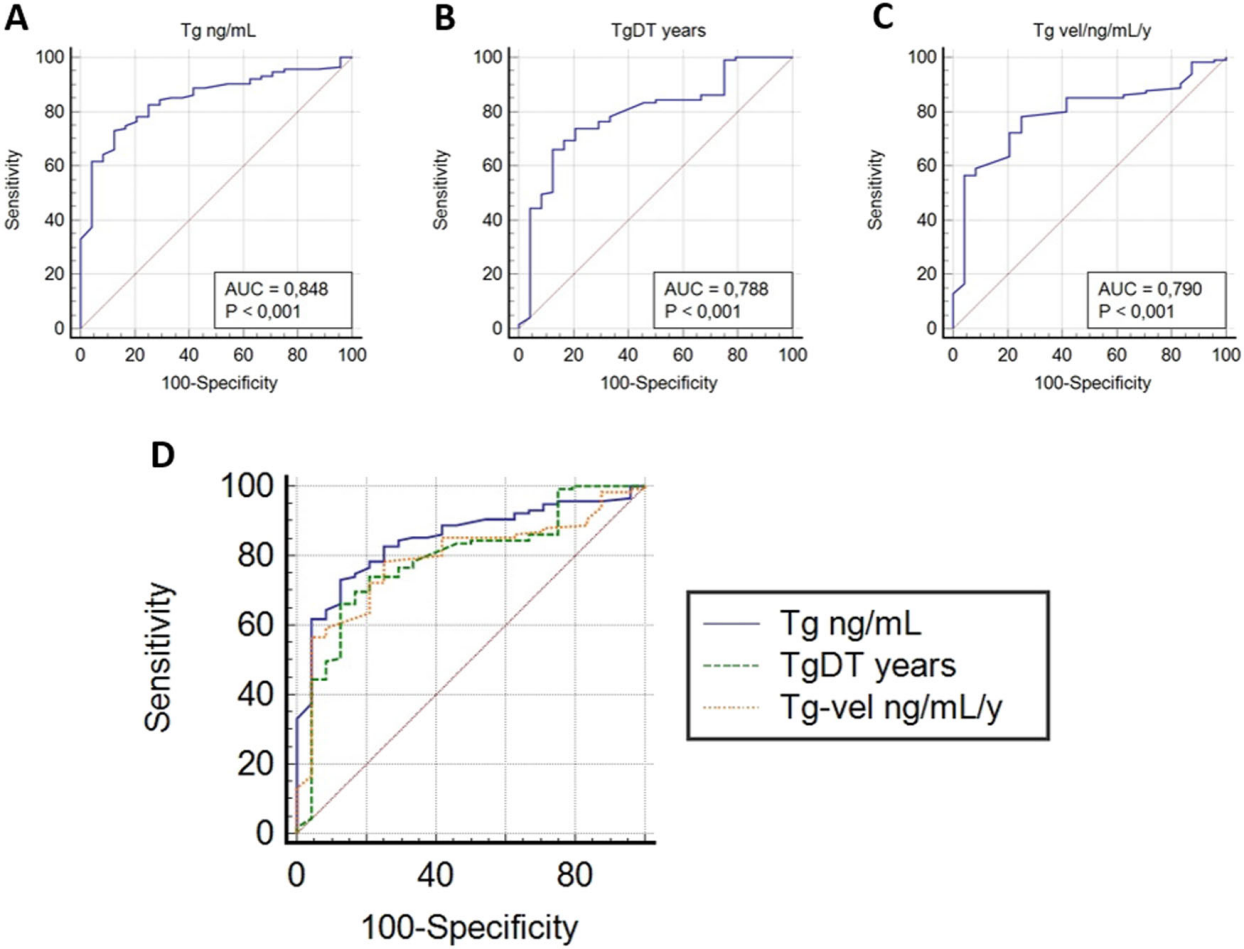
ROC curve analyses for the evaluation of the relationship between Tg in ng/mL (**A**), TgDT (**B**), and Tg-vel (**C**) thresholds for identifying positive PET/CT results. Comparison of ROC curves (**D**)

For the whole population, the ROC curves analysis (Fig. 1A–C) revealed the best stimulated Tg, TdDT, and Tg-vel thresholds of 18 ng/mL, 1.36 years and 1.95 ng/mL/y, respectively, to predict 2-[^18^F]-FDG PET/CT results (Table 2). The AUC of ROC curve for Tg was 0.058 greater than Tg-vel and 0.059 greater than TgDT but these differences were not statistically significant in both cases (*p* = 0.343 and *p* = 0.363) (Fig. 1D).

**Table 2:** Cutoff values calculated using ROC curve analysis considering the entire population

ROC curve analysis						
Parameter	AUC (95% CI)	*p* value	Cut-off value	Youden Index	Sensitivity	Specificity
Tg ng/mL	0.848 (0.778–0.903)	<0.001	18	0.605	73%	87.5%
TgDT y	0.788 (0.185–0.926)	<0.001	1.36	0.535	66%	87.5%
Tg-vel ng/mL/y	0.790 (0.713–0.854)	<0.001	1.95	0.532	78%	75%

### Relationship between the detection rate of PET and Tg kinetics in patients with Tg < 18 ng/mL

Dichotomizing our population using the Tg threshold of 18 ng/mL got from ROC curve analysis, the detection rate of 2-[^18^F]-FDG PET/CT in patients with stimulated Tg < 18 ng/mL (n = 52) was 60% due to the presence of increased FDG uptake sites in 31 cases; while in patients with Tg≥18 ng/mL (*n* = 87) the detection rate was 97% with 84 positive scans and only 3 negative.

**Fig. 2:**
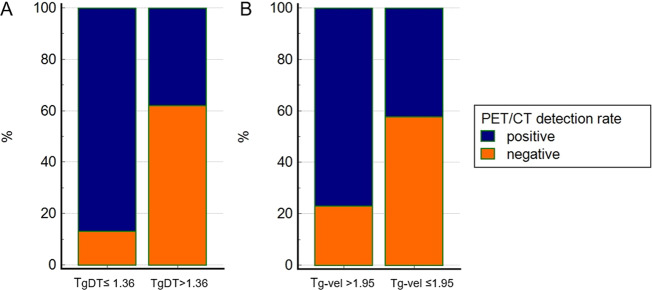
The relationship between PET/CT results and TgDT (**A**) and Tg-vel (**B**)

Focus on the group with Tg < 18 ng/mL, the detection rate of 2-[^18^F]-FDG PET/CT was significantly lower in patients with low Tg-vel (≤1.95 ng/mL/y) in comparison with high Tg-vel (42 vs 77%, p 0.018) and with long TgDT than short TgDT (≤1.36 years) (38 vs 87%, *p* = 0.001) (Fig. 2).

Subgroup with Tg ≥ 18 ng/mL was not further investigated due to the low negative PET/CT cases (*n* = 3) which limited the statistical analysis.

### OS

At a median follow-up of 44 months after PET/CT, death occurred in 43 patients (31%) with an average time of 50 months (range 2–173 months). The estimated 3-year and 5-year OS rates were 59% and 48%, respectively.

**Fig. 3:**
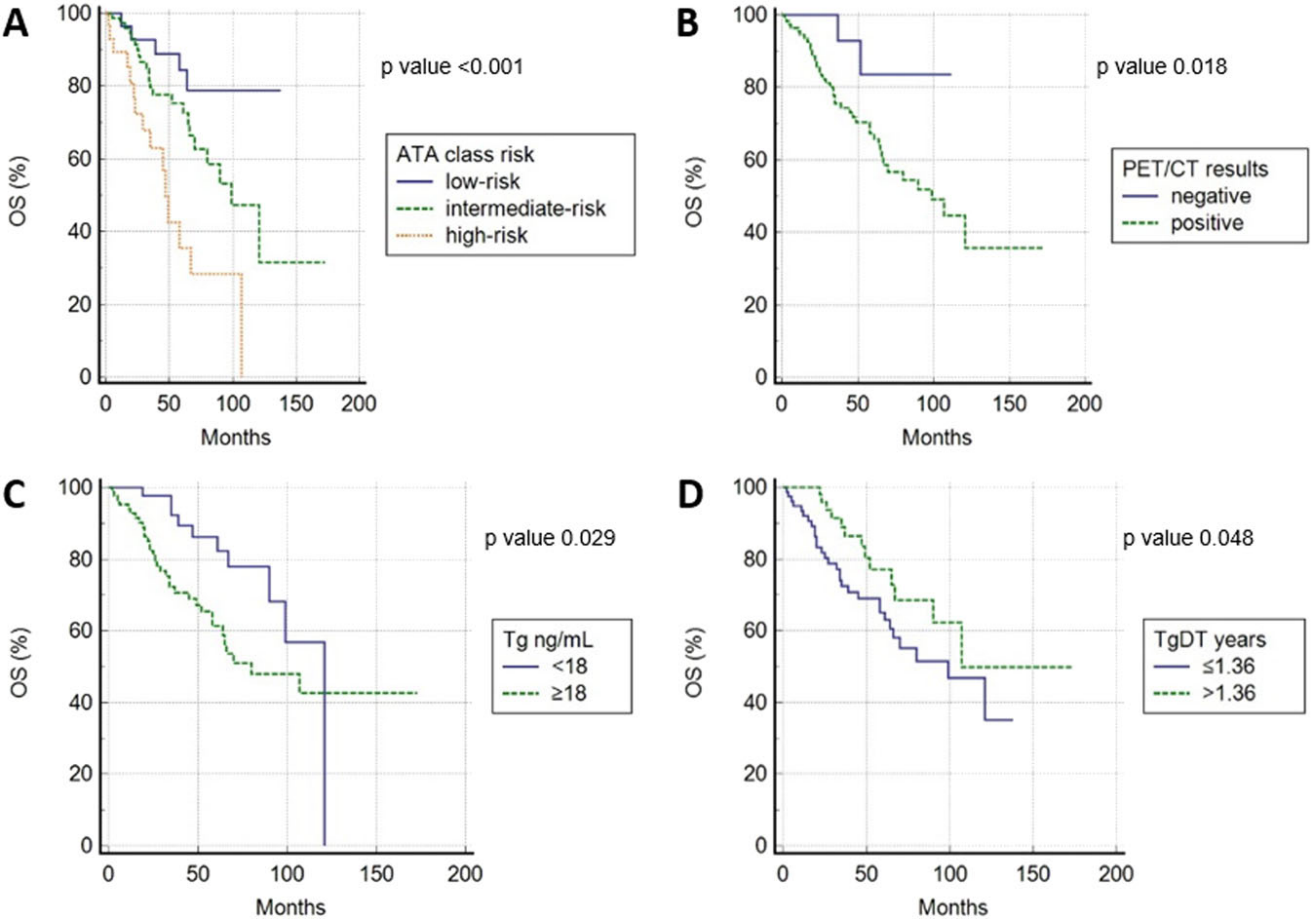
Overall survival curves according to ATA class risk (**A**), PET results (**B**), Tg before PET (**C**), and TgDT (**D**)

In univariate analysis, ATA class risk, 2-[^18^F]-FDG PET/CT results, stimulated Tg before PET, TgDT and the disease status at the last [^131^I]WBS before PET/CT (presence of bone metastases) were significantly correlated with OS (Fig. 3). Instead, the other clinical/pathological features and Tg-vel were not related with outcome survival. Also in multivariate analysis, ATA class risk, PET/CT results, Tg before PET and the disease status before PET/CT (presence of bone metastases) were confirmed to be independent prognostic variables for OS (*p* < 0.001, *p* = 0.036 and *p* = 0.014) (Table 3).

**Table 3:** univariate and multivariate analyses for OS

	Univariate analysis		Multivariate analysis	
	*p* value	HR (95% CI)	*p* value	HR (95% CI)
Gender	0.968	0.987 (0.540–1.806)		
Age	0.468	1.260 (0.673–2.358)		
Histotype	0.666	0.784 (0.239–2.494)		
Tumor size	0.885	1.084 (0.353–3.336)		
Multicentricity	0.997	1.002 (0.271–3.701)		
ATA class risk	<0.001	6.022 (2.335–15.530)	<0.001	3.233 (1.988–5.256)
Cumulative RAI activities administrated (GBq)	0.268	0.220 (0.069–1.903)		
N° radiometabolic therapies	0.272	1.897 (0.615–5.609)		
Tg at the time of ablation	0.164	0.090 (0.058–1.620)		
Presence of bone metastasis at ablation	0.099	0.150 (0.030–2.222)		
Positive PET/CT	0.018	0.422 (0.186–0.956)	0.036	1.803 (1.111–2.567)
Tg before PET/CT^a^	0.029	0.502 (0.272–0.935)	0.014	2.883 (1.236–6.722)
Tg-vel^a^	0.750	0.891 (0.437–1.861)		
TgDT^a^	0.048	0.583 (0.318–0.998)	0.055	0.501 (0.247–1.015)
Interval from diagnosis to PET/CT (years)	0.820	1.003 (0.972–1.035)		
Presence of bone metastases before PET/CT	<0.001	1.252 (1.111–1.500)	0.002	2.001 (1.250–3.108)

*OS* overall survival, *HR* hazard ratio, *CI* confidence interval, *N°* number

^a^Variables dichotomized using cutoff values after ROC analysis reported in Table 2

## Discussion

Tg is a protein expressed specifically by thyrocytes, both normal and pathological; for this reason, after thyroidectomy and [^131^I] ablation therapy it becomes an optimal marker for the detection of residual, persistent or recurrent disease [26, 27] and it is periodically measured. But, Tg measurement may be affected by several factors, such as the presence heterophile antibodies and anti-Tg antibodies [28], the dedifferentiation of DTC, TSH level [29] and the kind of assay method utilized [30]. Moreover, a single Tg measurement may not be exhaustive in the comprehension of disease status and treatment response, not considering intrinsically the previous measurements and the trend. Thus, other parameters which represent the Tg trend have been introduced, like TgDT and Tg-vel.

Some authors [14–16] demonstrated that TgDT was significantly associated with disease specific survival and their prognostic value was better than conventional variables, such as age and primary’s size. These authors underlined the idea that exponential changes in volume of growing tumoral cells may be related to Tg change, and TgDT could be only an indirect way to see this phenomenon.

Interestingly, the prognostic role of TgV was investigated only in one manuscript [23] showing significant impact for predicting DTC recurrence and OS, using a threshold of 0.3 ng/mL/y.

The combination of Tg and imaging examinations (ultrasound, [^131^I]WBS, PET,…) is absolutely fundamental in the management of DTC patients with suspected persistence of disease or relapse. Even more desirable, it would be to identify the right timing when to undergo 2-[^18^F]-FDG PET/CT, which is an expensive and associated with radiation exposure tool.

The detection rate of 2-[^18^F]-FDG PET/CT in studying DTC with detectable Tg and negative [^131^I]WBS is wide [8] and significantly associated with Tg value at time of PET scan. The last ATA guidelines [1] proposed a threshold of 10 ng/mL, but this value is not universally shared. For other cancers, like prostate cancer, serum marker kinetics (marker velocity or doubling time) seems to be more accurate than absolute value in predicting PET/CT findings and survival [31, 32]. The hypothesis of a connection between Tg kinetics and PET detection rate is logical and based on the idea that an aggressive and rapidly growing tumor should have a greater metabolic activity and is more likely to be FDG-avid. The effective usefulness of Tg kinetics in predicting PET/CT results is not completely understood with promising papers [21, 22]. Giovanella et al. [21] demonstrated that the accuracy of 2-[^18^F]-FDG PET/CT was significantly higher in patients with unstimulated Tg above 5.5 ng/mL and the TgDT below 1 year. A recent study [22] showed the superiority of TgDT using a threshold of 2.5 years compared to Tg as absolute value.

In our study, we analyzed and compared the diagnostic performance of all three Tg parameters (stimualted Tg before scan, Tg-vel and TgDT) in a large population of DTC with negative [^131^I]WBS. Applying ROC curve analyses in the whole population, we derived the best stimulated Tg, TdDT, and Tg-vel thresholds of 18 ng/mL, 1.36 years and 1.95 ng/mL/y which were confirmed to be significantly related with PET results and no factor showed to be better than others.

These results highlights that also Tg velocity may have a role in the definition of DTC aggressiveness, predicting PET/CT results.

Considering only patients with Tg < 18 ng/mL (a subgroup where the detection rate of 2-[^18^F]-FDG PET/CT was low with almost half of examinations negative for the recognition of disease), Tg kinetics showed to be useful in discriminating patient with positive scans. Both Tg-vel and TgDT significantly helped to predict PET/CT results.

With the help of TgDT (cutoff 1.36 years), the PET/CT detection rate passed from 38 to 87%; while using Tg-vel (cutoff 1.95 ng/mL/y) the PET/CT detection rate passed from 42 to 77%.

Further studies are needed to validate or controvert our results in a larger cohort and to identify potential new thresholds.

The approach proposed in our analyses, however, does not deal with the problem of serum Tg trend being unreliable in patients with anti-Tg antibodies [33]. Also anti-Tg antibodies change during the course of the disease, though their trend is not yet considered as a predictive factor.

About survival, we demonstrated that Tg before PET, PET results and ATA class risk were independent prognostic variables, while other Tg kinetics factors were not significantly related to survival. This evidence is partially in contrast with previous studies [14–16, 20], probably due to the heterogeneity of population investigated and the different timing of Tg kinetics measurement. Moreover, the heterogeneity of DTC evolution and disease status at time of PET/CT may influence this kind of evaluation. This is the first work where all Tg kinetics parameters (absolute value, velocity and doubling time) were investigated simultaneously.

Our study contains some limitations, like its retrospective nature, the potential use of heterogeneous management approaches over a relatively long period included, the absence of histological confirmation of all PET lesions and the difficult to analyze prognostic value of several variables specific of different timing of disease.

In conclusion, with this study we have demonstrated that Tg kinetics may help to predict 2-[^18^F]-FDG PET/CT results in DTC patients with negative [^131^I]WBS and detectable Tg, especially in case of low-moderate Tg. Moreover, stimulated Tg level before PET/CT is an independent prognostic factor for OS.
